# The Relevance of Thiamine Evaluation in a Practical Setting

**DOI:** 10.3390/nu12092810

**Published:** 2020-09-13

**Authors:** Federico Pacei, Antonella Tesone, Nazzareno Laudi, Emanuele Laudi, Anna Cretti, Shira Pnini, Fabio Varesco, Chiara Colombo

**Affiliations:** 1 ASST Nord Milano, UOC Neurologia, Ospedale Bassini, 20092 Cinisello Balsamo, Italy; 2 Department of Physical Rehabilitation, Casa di Cura Bonvicini, Via Michael Pacher 12, 39100 Bolzano, Italy; tesone.nutrizioneclinica@gmail.com (A.T.); emanuele.laudi@gruppobonvicini.it (E.L.); acretti.casadicura@gruppobonvicini.it (A.C.); shira.pnini@gmail.com (S.P.); f.varesco.casadicura@gruppobonvicini.it (F.V.); 3 Faculty of Medicine and Surgery, Medizinische Universitat Innsbruck, Christoph-Probst-Platz 1, Innrain 52 A, 6020 Innsbruck, Austria; nazzarenolaudi@gmail.com; 4 Lombardy Regional Course for General Practitioner, PoliS-Lombardia, Via Taramelli 12/F, 20100 Milano, Italy; chiara.colombo1981@gmail.com

**Keywords:** thiamine, thiamine deficiency, polineuropathy, Wernicke’s encephalopathy, wet beriberi, malnutrition, surgery, upper gastrointestinal surgery

## Abstract

Thiamine is a crucial cofactor involved in the maintenance of carbohydrate metabolism and participates in multiple cellular metabolic processes. Although thiamine can be obtained from various food sources, some common food groups are deficient in thiamine, and it can be denatured by high temperature and pH. Additionally, different drugs can alter thiamine metabolism. In addition, the half-life of thiamine in the body is between 1 and 3 weeks. All these factors could provide an explanation for the relatively short period needed to develop thiamine deficiency and observe the consequent clinical symptoms. Thiamine deficiency could lead to neurological and cardiological problems. These clinical conditions could be severe or even fatal. Marginal deficiency too may promote weaker symptoms that might be overlooked. Patients undergoing upper gastrointestinal or pancreatic surgery could have or develop thiamine deficiency for many different reasons. To achieve the best outcome for these patients, we strongly recommend the execution of both an adequate preoperative nutritional assessment, which includes thiamine evaluation, and a close nutritional follow up to avoid a nutrient deficit in the postoperative period.

## 1. Introduction

Thiamine, also called vitamin B1 or aneurin, is a water-soluble vitamin, and it is an essential micronutrient for human beings [1]. It is essential both as a coenzyme and noncoenzyme, and is involved in many processes, particularly in energy transformation, and oxidative and nonoxidative carbohydrate metabolism.

The human body is unable to synthesize thiamine, its supply relying almost entirely on dietary intake, even though some bacteria in the intestine can produce some small amounts of this vitamin [2].

Thiamine has a short half-life (1−12 h), and the body can store it for 1 to 3 weeks. Thus, a regular dietary supply is required to maintain correct thiamine levels [3]. Foods rich in thiamine are pork, poultry, eggs, fish (trout), legumes, nuts (macadamia), whole grains, cereals, seeds and yeasts. On the other hand, there are common food groups that contain low quantities of thiamine, such as polished rice, milled wheat flour, milk, vegetables and fruit [4,5]. Tea and coffee contain polyhydroxyphenols (tannic and caffeic acids) that break down ingested thiamine [5,6,7,8]. Moreover, many drug classes contain compounds that could alter the metabolism of thiamine:antacids; anticonvulsants, antineoplastic, contraceptives, diuretics are a few examples [9].

In many high-income countries, some foods are fortified with thiamine, contributing to about half of the required intake of this vitamin [10]. In low- and middle-income countries, the lack of dietary diversity and reliance on low-thiamine staple foods are the main causes of thiamine deficiency. High temperature and pH denature thiamine, thus processes such as cooking, baking, pasteurization and preserving foods can degrade thiamine [11]. Regular consumption of foods containing thiamine antagonists, for example betel nut or tea leaves, and thiaminases in foods such as raw fish and African silkworm larvae, is also implicated in the development of thiamine deficiency [12,13,14]. All these factors together explain why a relatively short period is sufficient for the development of thiamine deficiency and the appearance of its clinical signs [5].

Thiamine is absorbed by both active and passive uptake in the jejunum and ileum, mainly in the upper jejunum. The duodenum, ileum and the rest of the bowel play a less important role. After that, it is transported to the liver, to enter the red blood cells and/or white blood cells with a facilitative transport. These cells contain about 90% of the total thiamine in blood. The excess of thiamine, that is not bound to protein, is excreted through renal activity and the rate of its loss is strongly related to renal clearance [15,16]. Diuretics could be considered as the main cause of thiamine deficiency in cardiovascular patients [16].

The human intestinal thiamine absorption process is regulated through a molecular mechanism which involves the thiamine transporters-1 (hTHTR-1) and -2 (hTHTR-2) that are, respectively, the products of the Solute Carrier Family 19 Member 2 (SLC19A2) and SLC19A3 genes. Genetic disorders of thiamine transport and metabolism are a rare but treatable cause of thiamine deficiency that usually arises in childhood. Four genetic defects are reported: three with a predominantly neurological phenotype (SLC19A3, SLC25A19 and TPK1) and one with a multisystem disease (SLC19A2), including megaloblastic anaemia, thrombocytopenia, diabetes and hearing loss [17,18].

SLC19A2 dysfunction: SLC19A2 is a membrane thiamine transporter expressed in many human tissues, including the gastrointestinal tract. It encodes thiamine transporter 1 (THTR1), which facilitates thiamine transport across the cell membrane. *SLC19A2* homozygous mutations have been described as a cause of thiamine-responsive megaloblastic anemia (TRMA), an autosomal recessive syndrome characterized by megaloblastic anemia, diabetes and sensorineural deafness. *SLC19A2* deficit causes impaired insulin secretion, in conjunction with mitochondrial dysfunction, loss of protection against oxidative stress and cell cycle arrest. These findings link *SLC19A2* mutations to autosomal dominant diabetes and suggest a role of *SLC19A2* in β-cell function and survival [19].

SLC19A3 defect: Thiamine metabolism dysfunction syndrome type 2 is also known as: “*SCL19A3* gene defect”, “biotin-responsive basal ganglia disease” (BBGD) and “biotin-thiamine-responsive basal ganglia disease” (BTBGD). It is a potentially treatable disorder caused by mutations in the SLC19A3 gene, encoding the human thiamine transporter 2. The worldwide incidence and prevalence of this disorder are unknown. The clinical presentations of these syndromes are heterogeneous and related to the age of onset. They can be classified into three major categories: classical childhood BBGD, early-infantile Leigh-like syndrome/atypical infantile spasms and adult Wernicke’s-like encephalopathy [20]. Episodes of encephalopathy are caused by a substantially reduced capacity of mutant neuronal cells to increase SLC19A3 expression, necessary to adapt to stress conditions [21].

SLC25A19 defect: Thiamine metabolism dysfunction syndrome-4 (THMD4) represents an autosomal recessive ultrarare metabolic disorder characterized by childhood onset of episodic encephalopathy, often associated with a febrile illness, and causing transient neurologic dysfunction. Until now only two mutations (G125S and S194P) have been reported in the SLC25A19 gene as causative for this disease and a third mutation (G177A) as related to the Amish lethal microcephaly [22]. Brain imaging during the acute episodes shows lesions consistent with bilateral striatal degeneration or necrosis [17,23].

TPK1-related disease: TPK1 mutations are a rare, but potentially treatable, cause of thiamine deficiency. Thiamine pyrophosphokinase 1 (hTPK1, EC2.7.6.2) converts free thiamine to active thiamine pyrophosphate (TPP). Early recognition is, therefore, crucial given the potential benefits of thiamine supplementation. However, diagnosis of TPK1-related diseases is often delayed because of the clinical overlap with other metabolic diseases, including Leigh syndrome. TPK1-related disease presents recurrent episodes of postinfectious encephalopathy. Patients with this mutation could also experience epilepsy, learning difficulties, sensorineural hearing loss, spasticity and dysphagia. Consequently, patients often undergo numerous investigations, including muscle and skin biopsies, prior to receiving a molecular diagnosis [17].

Thiamine is found in four forms in the human body, free thiamine and three phosphorilated forms: mono-(ThMP), di-(ThDP) and triphosphate (ThTP) [5,24,25].

The metabolically active form, constituting 80% of total body thiamine is ThDP, which is also called thiamine pyrophosphate (TPP). ThDP is an essential cofactor in multiple enzyme complexes involved in oxidative decarboxylation, such as metabolism of glucose and branched-chain amino acids [12].

These enzyme complexes include the pyruvate dehydrogenase complex, the alpha-ketoglutarate dehydrogenase complex, and the branched chain alpha-keto acid dehydrogenase complex.

The most sensitive enzyme complex to thiamine deficiency seems to be the alpha-ketoglutarate dehydrogenase. The reduced activity of this enzyme complex can rapidly lead to reduced ATP synthesis, oxidative damage and, ultimately, cell death [26].

Thiamine circulates in erythrocytes and is delivered to cells with high metabolic requirements: brain, cardiac myocytes, liver, pancreas, skeletal and smooth muscles.

The recommended nutrient intake (RNI) of thiamine depends on gender and age: 1.2 mg/day for men and 1.1 mg/day for women, with an increase to 1.4 mg/day in pregnancy and 1.5 mg/day during lactation. In infancy, the adequate intake is set at 0.2 mg/day (0−6 months) and 0.3 mg/day (7−12 months). The RNI gradually increases to 0.5 mg/day for children aged 1−3 years, 0.6 mg/day for 4−6 years and 0.9mg/day for 7−9 years. After age 10, children’s thiamine requirement is the same as for adults. There are no known adverse effects of high thiamine intake, and there is no upper intake level for thiamine [27]. Listed in Table 1 there are some examples of food of common use with high content in thiamine.

## 2. Clinical Implications of Thiamine Deficiency

When healthy individuals are deprived of thiamine, thiamine stores are depleted within 1 month (usually 2 weeks). However, 1 week without thiamine intake can lead a healthy person to develop a resting tachycardia, weakness and decreased deep tendon reflexes. Peripheral neuropathy can arise too. Thiamine deficiency could be divided into two major clinical pictures: dry beriberi (with nervous system involvement) and wet beriberi (with cardiovascular involvement).

Diseases caused by thiamine deficiency were first recorded in China in the 10th century. In Japan, it was called “kakkè”. Japanese military were affected by wet beriberi because their diet was mainly based on polished rice, which is very low in thiamine [30].

### 2.1. Dry Beriberi, Wernicke’s Encephalopathy and Other Neurological Conditions

Thiamine deficiency could lead to several neurological complications, which can be life threatening, if not promptly recognized. It can affect both the central and peripheral nervous systems.

#### 2.1.1. Wernicke’s Encephalopathy

Wernicke’s encephalopathy (WE) is an acute neurological disorder caused by thiamine deficiency. WE is usually described in alcohol abuse, especially chronic, but it is associated also with prolonged fasting, malnutrition, malabsorption, gastrointestinal malignancies, dialysis, AIDS, gastrointestinal surgery and bariatric surgery [31,32,33,34,35]. The classic triad of symptoms in WE is ophthalmologic dysfunction and nystagmus, ataxia and mental status changes. The European Federation of Neurological Societies (EFNS) [36,37] developed guidelines for the diagnosis, management and prevention of Wernicke’s Encephalopathy, which must include 2 signs out of 4 of the following: (1) dietary deficiencies, (2) visual impairment, (3) cerebellar dysfunction and (4) either an altered mental status or mild memory loss. Wernicke’s Encephalopathy is a life-threatening condition, but it can be prevented and treated if promptly recognized.

#### 2.1.2. Korsakoff’s Syndrome

Korsakoff’s syndrome (KS) can develop in patients who previously suffered from Wernicke’s encephalopathy, but did not receive immediate and adequate treatment with thiamine replacement therapy [38]. KS is probably always preceded by WE [39]. This uncertainty arises because the clinical diagnosis of WE is very difficult and often does not receive adequate treatment. Korsakoff’s Syndrome symptoms include mainly global amnesia, but in more severe cases, there can be cognitive and behavioral dysfunctions, such as confabulations, apathy, affective disorders, and alteration of emotion, perception and social cognition [40].

#### 2.1.3. Marchiafava-Bignami Syndrome

Marchiafava–Bignami disease (MBD) was originally described as a rare, fatal disease affecting wine drinkers [41]. It has been long considered to have a toxic or nutritional aetiology, and it is characterized by the demyelination and necrosis of the corpus callosum [42]. Chronic patients with alcohol use disorder are often affected by malnourishment and vitamin deficiencies, mainly regarding vitamin B1. This may lead to MBD, due to liver damage-induced metabolic disturbance ultimately leading to a B1 hypovitaminosis [43]. The clinical picture could be very variable: altered mental state, pyramidal signs, signs of disconnection, split brain syndrome, primitive reflexes, sensory symptoms, gaze palsy or diplopia.

#### 2.1.4. Alzheimer’s Disease (AD)

Alzheimer’s disease is defined as a cognitive decline with typical neuropathological modifications characterized by neurofibrillary tangles (NFTs) and amyloid plaques [44,45]. In recent years, a large amount of evidence has arisen, highlighting the possible relation between thiamine deficiency as one of the possible underlying mechanisms in AD [46]. The main mechanisms that are considered as causative for this relationship are:

(1) Thiamine facilitates neurotransmission: the more accreditated mechanism for this activity is the potentation of the release of neurotransmitters such as dopamine [47], achetylcholine [48] and norepinephrine [49].
(2) Glucose metabolism: it is diminished both in AD patients and in those affected by thiamine deficiency. The metabolism of glucose in the brain is very high and it requires thiamine for critical processes. The sensitivity of the brain to thiamine deficiency could be explained by the fact that the human brain represents 2% of the body mass, but it consumes about 20% of the total glucose intake. Additionally, glucose in the brain is a substrate for the synthesis of neurotransmitters such as acetylcholine and glutamate [50,51,52]. Thiamine acts also as coenzyme activities for the mitochondrial enzymes [alpha]-ketoglutarate dehydrogenase and pyruvate dehydrogenase, both involved in glucose metabolism.
(3) Thiamine-dependent processes are reduced in AD patients. In studies, this reduction varies between 50% and 100% [53,54].
(4) Studies in animal models demonstrated that thiamine deficiency could play an important role in AD pathophysiology. It produces deficit in the cholinergic system [55], induces excess glutamate release and selective cell death in the submedial thalamic nucleus [56,57,58], exacerbates the formation of the plaques and also increases the phosphorylation of tau [59,60].
(5) The effect of the reversal of thiamine deficiency, especially with the administration of benfotiamine, on cognitive impairment was evaluated in animal studies with the evidence of a clear improvement in cognitive performance and reduction in phosphorilated tau and plaques formation [61,62].
(6) In the brain, thiamine acts also by binding prions [63], as antioxidant [64] and interferes with acetylcholine release [58].

In view of the above, long term thiamine deficiency could represent a key factor for the formation of NFTs and plaques, thus representing a pathogenic mechanism in AD [46].

#### 2.1.5. Depression

Thiamine is involved in the synthesis of different neurotransmitters, for example serotonin, aspartate, glutamate and acetylcholine. It is not clear in which way depression and thiamine deficiency are linked, but it is known that low levels of these neurotransmitters could lead to depression [65]. Thiamine deficiency is also involved in oxidative stress [66], which is related to a reduction in hyppocampal volume and neuronal damage in depressed patients [67]. Different studies in literature show that low thiamine levels are associated with a higher prevalence of symptoms of depression [68,69]. Despite these data, the effects of thiamine supplementation on depression is not well established. Further studies are needed to clarify this possible association.

#### 2.1.6. Polineuropathy

Peripheral neuropathy is characterized by the symmetrical impairment of sensory, motor, and reflex functions of the extremities, affecting mainly the distal lower limbs. Histologic analysis shows a degeneration of the myelin in the muscular sheaths, which cause lesions, with no inflammation [70]. The main electrophysiologic findings were those of axonal neuropathy, most prominent in the lower limbs. Axonal degeneration is also found in sural nerve biopsy specimens. Subperineurial oedema was commonly observed.

### 2.2. Wet Beriberi

Cardiovascular signs of thiamine deficiency could be divided into two forms: chronic form and acute fulminant form, also known as Shoshin beriberi [71]. Wet beriberi, characterized by high cardiac output with predominantly right-sided heart failure and lactic acidosis, is rarely seen in modern society [72].

#### 2.2.1. Chronic Form

This form consists of 3 phases. The first phase is characterized by peripheral vasodilatation that leads to a high cardiac output state. The consequences are salt and water retention, mediated through the renin-angiotensin-aldosterone system in the kidneys. In the second phase, as the vasodilatation progresses, the kidneys detect a relative loss of volume and respond by conserving salt. This also determines the absorption of fluid in the circulatory system, resulting in an excess that leads to oedema of the extremities.

The third phase includes a significant oedema, and the heart is exposed to a very high workload to pump the required cardiac output. This determines an overuse injury to parts of the cardiac muscle, which results in the physical symptoms of tachycardia, oedema and high arterial and venous pressures. These changes can lead to myocardial injury, expressed as chest pain [71].

#### 2.2.2. Shoshin Beriberi

Shoshin beriberi, or acute fulminant cardiovascular beriberi, is a faster form of wet beriberi. Shoshin beriberi, an uncommon cause of hemodynamic instability (or cardiac shock) and acute heart failure, may be undiagnosed in Western countries because of its low prevalence. This severe heart condition, due to thiamine deficiency, is rapidly fatal unless a specific therapy is given. The main organ which is affected is the heart, which is unable to satisfy the body’s demands because of a rapid deterioration of the muscle. Acute heart failure due to Shoshin Beriberi does not show specific signs on the electrocardiogram. The chest x-ray simply shows signs of pulmonary oedema and heart enlargement, and oedema may not be present. The echocardiography may be normal although hypokinesia and/or dilatation of the left ventricle (due to thiamine deficiency) are sometimes noted. Cyanosis of the hands and feet, tachycardia, distended neck veins, restlessness and anxiety are observed. Clinical improvement is rapid after intravenous infusion of vitamin B1, and support of the heart function is required at this stage. Recovery is usually swift and complete if treatment is initiated promptly. However, if no treatment is available, death occurs rapidly [72].

## 3. Other Clinical Conditions Related to Thiamine Deficiency

For many years, thiamine deficiency was referred to in the context of beriberi.

This perception has been changing and the interest in thiamine deficit as a risk factor for different systemic diseases has been increasing [73,74,75].

### 3.1. Diabetes Mellitus (DM)

The link between DM and thiamine deficiency is not clearly understood. The complications of thiamine deficiency in DM include hyperglycaemic induced cellular damage, endothelial dysfunction and increased oxidative stress. The reciprocal association between insulin and thiamine could somehow answer the questions: 1) thiamine increases insulin sensitivity; 2) thiamine deficiency leads to severe dysfunction in insulin synthesis and secretion [4]. Diabetic nephropathy affects particularly the proximal tube where reuptake of thiamine occurs, so thiamine deficiency could be attributed to the increased renal clearance due to DM [76]. Additionally, intestinal motility is affected by DM’s autonomic neuropathy with the promotion of overgrowth of intestinal bacteria with the consequence of a reduction in thiamine absorption [77]. Thiamine supplementation in diabetic patients demonstrates different advantages such as the regression of microalbuminuria and decreased plasma fasting glucose concentration [78,79,80]. Further research in the field of the efficacy of thiamine supplementation in diabetic patients is needed.

### 3.2. Immune System

Thiamine has antioxidant effects on neutrophiles, a protective role in macrophage suppressing oxidative stress-induced activation of NF-kB, as well as a central role in the activity of p53 suppressor protein with the inhibition of p43 intracellular activity and affects the release of a specific member of the intracellular adhesion molecule [68].

### 3.3. Endothelial Function

Thiamine and its derivates act as anti-oxidants and can improve endothelial function [81]. These benefits are seen in both euglycemic and hyperglycaemic patterns. Loss of arteriovascular resistance was seen in patients with clinical thiamine deficiency [82]. In literature, thiamine supplementation showed a decrease in systemic vascular resistance [83]. Thiamine supplementation also demonstrated the ability to restore endothelial function in patients with smoking induced endothelial dysfunction [78]. At the moment, there is great evidence that chronic vascular inflammation and its consequent dyslipidemia are inversely associated with thiamine levels [72].

### 3.4. Cerebrovascular Diseases

Different case reports have suggested a potential role of thiamine deficiency in hemorrhagic and ischemic cerebrovascular events [84,85,86,87]. The exact mechanism that links thiamine with the brain is not fully understood, and it is probably related to thiamine ability to improve endothelial function.

## 4. Thiamine Supplementation and Other Nutrients

There are three ways to administer thiamine to a thiamine deficient patient: oral, intravenous, or intramuscular.

The oral administration should be preferred in outpatient settings or if the patient does not have the possibility to organize an intravenous administration or cannot undergo an intravenuos access procedure.

There is not a unique suggestion about dosage and duration of thiamine supplementation. For the treatment of early symptoms of neuropathy, some authors suggest an oral intake of 20–30 mg/day, until the disappearance of symptoms [88]. Other authors suggest higher dosage and higher frequency, 100 mg twice or thrice a day [89,90].

We do not recommend oral administration, since we consider it to be not adequate for the treatment of symptomatic patients. Moreover, gastrointestinal surgery patients have altered structure and function possibly leading to an impaired thiamine absorption.

Intravenous (IV) administration should be regarded as the best option and must be considered for the inpatient approach. For patients with mild symptoms, authors suggest 100 mg thiamine IV for a period of 7–14 days based on symptoms [91]. For patients with severe thiamine deficiency, such as Wernicke’s Encephalopathy, the suggested dosage ranges from >100 mg IV for several days, followed by IM or oral intake of high doses until the resolution or clear improvement of the symptoms [92], to 500 mg IV thrice a day for 3–5 days followed by 250 mg/day for the same period or until the resolution of the clinical picture. It is suggested to take into consideration the treatment with oral formulation of 100 mg/day until the risks factors have been managed or indefinitely [88,90].

Intramuscular supplementation should be taken in consideration only of those patients without an IV access and in emergency situations [89]. A dosage of 250 mg IM for 3–5 days is suggested for the treatment of WE patients’ secondary alcoholism [93]. Some authors have used the same regimen for the treatment of WE after bariatric surgery [89].

Magnesium must be monitored, as it is a cofactor for the metabolism of TPP, and if it is lower than normal, it must be normalized [94]. In its guideline for the management of WE, the Royal College of Physicians suggests that, to every 250 mg of thiamine, there should be an addition of 4 mg of vitamin B2, 50 mg of Vitamin B6, 160 mg B3, 500 mg Vitamin C, 60–180 mEq potassium, 10–40 mmoL/L phosphate and 10–30 mEq magnesium [95]. It is important to underline the urgency of the intervention as delay could put the patient at risk for death or severe and irreversible damage [96].

## 5. Neuroimaging

The most typical neuroimaging finding of thiamine deficiency is the one present in Wernicke’s encephalopathy. Cytotoxic and vasogenic oedema are the most frequent alterations [97].

The derangement of ionic flow through the cell membrane, due to the alterations in thiamine-related glucose and oxidative cellular energy metabolism, causes an intracellular water-shift and a consequent cell injury (cytotoxic oedema). These alterations also interfere with the blood-brain barrier permeability, determining the penetration of intravascular fluids in the extracellular space (vasogenic oedema) [98,99].

A CT scan is not a recommended test for WE although it could detect, in the subacute and chronic stages, low density abnormalities in both typical and atypical sites expression of oedematous lesions [100,101].

MR imaging has a great advantage over CT, both in visualizing and quantifying this type of lesion. In fact, they appear as signal hyperintensities on T2-weighted images, due to the water content of the lesions.

The typical lesions are bilateral and located in the thalami, mamillary bodies, tectal plate (superior and inferior colliculi) and periacqueductal area. These regions are probably more sensitive to thiamine deficit because of their particularly high rate of thiamine related glucose and oxidative metabolism [102]. Atypical lesions are instead located in the vermis, cerebellum, cranial nerve nuclei, dentate nuclei, red nuclei, splenium and cerebral cortex, in particular in the frontal and parietal cortices [97,98,100,101,103]. Generally, atypical MRI findings accompany typical findings. The cortical involvement was proposed as an indicator for poor prognosis [104].

The specificity and sensitivity were 93% and 53%, respectively, and the positive predictive value was 89% [100]. Conventional MRI can show lesions in nearly two-thirds of the subjects with a clinically verified acute WE. Additional information could be added with the use of fluid-attenuated inversion recovery (FLAIR) images and diffusion-weighted imaging (DWI) [105,106,107]. The use of an intravenous contrast agent, such as gadolinium, could point out signal abnormalities in affected regions. However, this gadolinium contrast enhancement can be absent in the acute stage, when cytotoxic oedema anticipates the alteration of the blood-brain barrier (BBB) and the consequent vasogenic oedema [97,108].

MR spectroscopy shows low N-acetylaspartate/creatinine ratio (NCC/Cr) as expression of neuronal impairment, and a high lactate peak [109]. The NAA/Cr improves in parallel with clinical improvement following thiamine therapy [110]. This progress is also seen in the other MRI sequences up to a complete resolution of the abnormal signals after the introduction of thiamine supplementation and in parallel with the amelioration of the clinical condition.

Different authors suggest that the distribution of the lesions is different in patients with or without a story of alcohol abuse. In particular, atypical lesions are more easily seen in nonalcoholic patients [103,104,111].

The heterogeneity of MRI lesions could depend on the severity of the disease, the timing in which the exam was carried out and the acuteness of the disease. In literature, there is not a clear indication regarding which is the best MRI technique to use. We think that T2-weighted imaging and FLAIR sequences are the most useful to identify the tissue alterations. We also think that MRI is a very powerful tool in the hands of clinicians, and it should be used to support the diagnosis of WE that remains primarily clinical, both in alcohol and nonalcohol user patients. We suggest performing an MR investigation in every suspected WE patient, but the execution of this exam should not delay the beginning of a thiamine supplementation when the clinical picture suggests a WE. We do not recommend the use of a CT scan for the diagnosis of WE, but we think that it should be performed in an emergency setting to exclude other mimicking conditions.

The neuroimaging findings of Korsakoff’s Syndrome should be evaluated in the context of the brain insult caused by chronic alcoholism itself, as Korsakoff’s syndrome occurs especially in patients with a history of alcohol abuse and it is always preceded by an episode of WE: the most frequent alteration is volume deficits in the same areas of Wernicke’s encephalopathy findings [112]. So, MR shows ventricle enlargement, aqueductal dilatation, cortical thinning and sulcal widening [113].

The radiological characteristics of Marchiafava-Bignami disease (MBD) are lesions located across the entire corpus callosum independently of the timing in which the brain imaging was performed (acute or chronic phase of the disease). Lesions frequently persist during follow up. Among MBD patients, callosal atrophy and/or necrosis was frequently reported, and also gadolinium contrast enhancement of the lesions was frequently present [42].

## 6. Risk of Thiamine Deficiency in Upper Gastrointestinal (GI) Surgery Patients

Table 2 shows the causes of thiamine deficiency, ranging from poor intake to poor absorption, increased loss and increased utilization (Table 2). Though not common, the overgrowth of small bowel bacteria could lead to low thiamine levels [114] and should be taken into account in surgical patients’ Upper GI surgery; candidate patients can develop thiamine deficiency for many different reasons.

Several of these mechanisms can be found in patients with upper GI problems. Obese patients can develop thiamine deficiency due to malnutrition, malabsorption syndrome and because of gastric bypass surgery complications. Additionally, obese patients may develop thiamine deficiency due to their diet, highly caloric and rich in simple carbohydrates, requiring increased levels of thiamine utilization and ultimately leading to thiamine deficiency [115]. Oncologic patients, before and after surgery, can develop Vitamin B1 deficiency because of hyperemesis, malnutrition, malabsorption but also due to antineoplastic drugs. The nutrition support clinician should be aware of patients who may be at risk for thiamine deficiency. Risk factors include 1 or more nutrition-related aetiologies: decreased nutrient intake, increased nutrient losses or impaired nutrient absorption.

**Table 2:** Causes of thiamine deficiency [116].

Causes of Thiamine Deficiency			
Poor Intake	Poor Absorption	Increased Loss	Increased Utilization
Diets primarily high in polished rice/processed grains	Malnutrition	Diarrhea	Pregnancy
Chronic alcoholism	Gastric bypass surgery	Hyperemesis (gravida rum or not)	Lactation
Parental nutritional without adequate thiamine supplementation	Malabsorption syndrome	Diuretic use	Hyperthyroidism
Gastric bypass surgery		Renal replacement therapy	Refeeding syndrome

In literature, there are many papers describing the association between upper GI surgery and complications related to thiamine deficiency [30,31,32,33,34,117,118,119,120,121,122,123,124,125].

Independently, oncologic patients, obesity patients or other patients that undergo surgery of the upper GI tract are exposed to the risk of development of thiamine deficiency. Despite this, it is possible that many practitioners have never seen complications related to thiamine deficiency.

All these patients, independently of the underlying condition, can experience symptoms such as unrelenting vomiting, rapid weight loss before and/or after surgery (about 30–40 kg in 3 months) [35,126] and malnourishment. Recurrent vomiting, excessive weight loss and malnutrition are perhaps the main causes for depletion of the vitamin reserve.

Moreover, patients that are on nutrition support (e.g., obese patients undergoing a strict dietary regimen or patients unable to take the correct amount of calories for different reasons) might experience thiamine deficiency and its related complications.

Small-bowel feeding could impair thiamine intake due to the bypass of the preferred absorption site [87]. Often, surgical patients in the days before and/or after surgery are on artificial enteral or parenteral nutrition. This could lead to a thiamine deficit, if it is not well balanced and checked by a nutritional specialist.

Nowadays, there are no indications that suggest the evaluation of thiamine concentration in surgical patients before or after the procedure. We strongly recommend measuring thiamine plasma concentration before surgery in every patient who may be at risk of thiamine deficiency, starting a replacement therapy when needed. If the patient is not hospitalized, this could be started with intramuscular administration to avoid any absorption problems. The dose and duration of the therapy should be customized, also taking into account the morbid condition that led to the deficit and the low plasma levels. Patients that have any of the abovementioned symptoms must be considered at a higher risk of developing thiamine deficiency and its complications and must be strictly monitored. The period after surgery must be considered at higher risk for the development of thiamine deficiency. If one or more of the clinical pictures appear we suggest a rapid and strong therapy. For Wernicke’s encephalopathy, in consideration of our experience in the treatment of this type of condition both in patients with chronic alcohol use and without alcohol use, we suggest a starting dose of 500 mg IV thrice a day for the first three or five days in consideration of the severity of the clinical picture and the underlying cause that generates the deficiency. We also suggest, as a follow-up measure and as already reported in literature, a 250 mg/day IV for an additional three days then an oral, if possible, or intramuscular administration for a long period or until the patient can achieve proper nutrition. We always suggest using a higher dosage also because no cases of thiamine toxicity have been reported from the use of thiamine at the dosages indicated, even in patients in critical condition [127].

Considering the mortality and morbidity that can be caused by thiamine deficiency, we suggest including its measurement in the tests when staging the patient and his morbid condition, especially if symptoms such as vomiting are present. Each clinical picture discussed in the paper is easily preventable by thiamine administration, if it is diagnosed before symptoms appear. We would like to emphasize that neurological complications, except for polineuropathy and wet beriberi, are life-threatening conditions that could be prevented and successfully treated if promptly recognized. Polineuropathy could negatively impact the quality of life of the patient. Additionally, the conditions related to a chronic thiamine deficiency, Alzheimer’s disease, diabetes and vascular problems among others, must be taken in consideration in the long term for patients that underwent upper GI surgery. Therefore, we suggest a life-long monitoring of thiamine plasma levels.

We would also like to underline that in such patients, thiamine deficiency might be associated with low levels of other micronutrients and electrolytes, thus further complicating the clinical picture.

Refeeding syndrome is one of these conditions that must be always kept in mind as it could be present in patient on parenteral nutrition, and also thiamine deficiency is a hallmark manifestation of the syndrome with hypophospatemia [128,129,130,131]

Physicians must be aware of these conditions, their management and, in general, of all the warning signs shown by patients with a higher risk of developing a thiamine deficiency condition.

It is also very important for patients to be informed of every sign and symptom that could potentially lead to thiamine deficiency and must be instructed to promptly inform their physician if any of these appear. In any case, in consideration of the high tolerability of the treatment and the absence of toxicity, we suggest starting thiamine administration immediately if a patient of possible thiamine deficiency exists.

In this way, it is possible to promptly recognize and treat this condition before the appearance of a definite clinical picture [124].

## Figures and Tables

**Table 1:** List of foods with a high thiamine (vitamin B1) content, from the richest to the least rich. The content of thiamine (vitamin B1) refers to 100 g of edible part of each food [28,29]. RDA: Recommended Daily Allowance.

FOOD	THIAMINE MG	% RDA
Kellogg’s all-bran	2.27	162.14%
Wheat germ	1.882	134.43%
Sunflower seeds	1.48	105.71%
Karkadè	1.279	91.36%
Macadamia nuts	1.195	85.36%
Parma Ham	1.03	73.57%
Beans	0.9	64.29%
Lentils	0.873	62.36%
Oats	0.763	53.86%
Wurstel	0.593	42.36%
Whole wheat pasta	0.488	34.86%
Bread	0.473	33.79%
English muffins	0.431	30.79%
Pork Ribs	0.418	29.86%
Bagel	0.403	28.79%
Quinoa	0.36	25.71%
Hamburger	0.349	24.93%
Cashew butter	0.312	22.29%
Green Beans	0.3	22%
Blue fin tuna	0.241	17.21%
Pizza	0.211	15.07%
Asparagus	0.121	8.6%
Nori seaweed	0.098	7%
Parsley	0.086	6.14%
Tofu	0.081	5.79%
Spinach	0.078	5.57%
Broccoli	0.071	5.07%
Carrots	0.066	4.71%
Fried chicken	0.051	3.64%
Onion	0.046	3.29%
Egg	0.04	2.86%
Lobster	0.02	1.43%
Soy sauce	0.004	0.29%
Marshmallow	0.001	0.07%
